# Biological incorporation of human acellular dermal matrix used in Achilles tendon repair

**DOI:** 10.1007/s10561-017-9628-3

**Published:** 2017-04-28

**Authors:** Giampietro Bertasi, Windy Cole, Brian Samsell, Xiaofei Qin, Mark Moore

**Affiliations:** 1 University of Padua, Padua, Italy; 2 Robinson Wound Care Center, 1533 South Water Street, Kent, OH 44240, USA; 3 LifeNet Health, 1864 Concert Drive, Virginia Beach, VA 23453, USA

**Keywords:** Acellular dermal matrix, ADM, Allograft, Matracell, Tendon augmentation, Histology

## Abstract

Human acellular dermal matrices (ADMs) are used successfully in a variety of procedures, including sports medicine related, wound repair, and breast reconstructions, but the mechanism of repair is still not fully understood. An opportunity to explore this mechanism presented itself when a patient experienced a rerupture of the native tendon due to a fall that occurred 2 months after undergoing an Achilles tendon repair using Matracell treated ADM. The ADM was removed and an extensive histology analysis was performed on the tissue. Additionally, a literature review was conducted to determine the mechanism of ADM integration into the tendon structure and explore if differences in this mechanism exist for different types of human ADMS. The histology analysis demonstrated that the healing process during a tendon reconstruction procedure is similar to that of wound healing. Furthermore, the literature review showed that differences exist in the mechanism for integration among various human ADMs and that these differences may be due to variances in the methods and technologies that manufactures use to process human ADMs.

## Introduction

The use of matrix-derived biologic scaffolds has become a frequently used option in a variety of clinical applications though not all matrix types respond similarly. Xenografts may still contain alpha-gal, an epitope not present in humans, even after decellularization, leading to negative interactions with human anti-alpha-gal-antibodies (Galili 1993, 2001). Furthermore, there have been potential immunogenic issues noted with decellularized porcine small intestine submucosa (SIS) (Bellows et al. 2006; Zheng et al. 2005), even leading some surgeons to discontinue xenograft SIS use (Iannotti et al. 2006). Synthetic matrices, another option, can be difficult to create with the same make-up and orientation as naturally occurring extracellular matrices (Cheng et al. 2014). In contrast, human ADMs have been successfully used in a wide variety of procedures including wound healing, soft tissue repair, and sports medicine applications (Bullocks 2014; Levenda and Sanders 2015; Walters et al. 2016). Theoretically, the decellularization process removes potentially immunogenic cellular constituents yielding a biocompatible scaffold that can be integrated by host cells (Norton and Babenesee 2009). However, there are differences at the cellular level in how these human ADMs integrate with the host. These differences in the individual mechanism of integration for each ADM type are not well understood. Furthermore, obtaining histological data on incorporation can be difficult unless a case presents itself where a second surgery becomes necessary. During the second procedure there may be an opportunity where biopsies can be taken of the ADM.

ADMs can be especially useful in tendon augmentation. The graft can offer mechanical support while also improving the healing process by influencing host cell infiltration (Aurora et al. 2007). The mechanical support provided by the ADM reduces the risk of re-injury of the tendon in repair procedures such as rotator cuff repair and the management of Achilles tendon ruptures (Barber et al. 2012; Tezeren and Kuru 2006). The use of ADMs to augment tendon repair can also provide an additional advantage of reducing the complexity of the surgical procedure by removing the need for a second surgical site for the recovery of autologous donor tissue and thus eliminating the risk of second site morbidity (Lee 2008). Important qualities to consider when selecting an ADM type include incorporation ability and easy intraoperative handling (Lee 2008).

This paper will provide a histologic analysis on human dermis processed with Matracell^®^ and Preservon^®^ technologies, and hereafter referred to as M-ADM. M-ADM, under the trade-names DermACELL^®^, ArthroFlex^®^, and OrACELL^®^, has been successfully used to heal burn wounds, close diabetic foot ulcers, enable successful breast reconstructions, aid in tendon repairs, and provide barriers for guided bone regeneration (Bullocks 2014; Chen et al. 2012; Levenda and Sanders 2015; Wallace 2013; Walters et al. 2016). M-ADM is rendered acellular using non-denaturing anionic detergent, recombinant endonuclease, and antibiotics whereby ≥97% of DNA from the tissue is removed (Moore et al. 2015). Terminal sterilization with a Sterility Assurance Level (SAL) of 10^−6^ is achieved through the use of a controlled, low dose of gamma irradiation applied at low temperature (Moore et al. 2015; Samsell and Moore 2012). M-ADM also exhibits ease of handling with the ability to be stored fully hydrated at ambient temperatures using glycerol preservation technology (Moore et al. 2015).

While the mechanism which allows ADMs to integrate with host tissue is not fully understood, this report aims to provide a greater understanding of how the ADM scaffold is incorporated in tendon repair. In this histological case study, a patient underwent primary Achilles tendon repair with M-ADM augmentation between the tendon and paratenon. The M-ADM graft was removed 2 months post-operative following re-rupture of the native tendon due to a fall and an extensive histology analysis was conducted on the integrated M-ADM.

The second surgery following re-injury provided a fortuitous opportunity to explore the remodeling composition of the ADM following augmented tendon repair. Furthermore, a literature investigation was undertaken to determine how ADMs incorporate into the tendon structure and what mechanism could be responsible for influencing the strength of repair. These results are presented here.

## Case notes

A 35-year-old patient underwent an end-to-end primary repair of Achilles tendon rupture. The severed ends of tendon re-approximated with sutures and M-ADM (DermACELL, LifeNet Health, Virginia Beach, VA) was placed between the tendon and paratenon to augment the repair. The post-operative course was unremarkable and the surgical site healed well. At 2 months post-operative, the patient fell and re-ruptured his Achilles tendon. The re-rupture occurred at the primary repair site and surgical intervention was necessary to address the injury. Approximately 1 month after the re-rupture, the area was surgically opened and histology specimens were obtained from the ADM-paratenon interface. Upon removal, it was noted that the ADM had adhered to the host tissue and that the rupture had transected both the host tendon and M-ADM graft. At this revision surgery, M-ADM was again used to augment the repair. The patient’s post-op progress was satisfactory and the repair successful.

## Histology analysis

Multiple sections of explanted tissue were prepared for histological evaluation. The specimens were taken from the area of Achilles tendon sutures. The histology slides were prepared and analyzed by the Biology Lab at the University of Padua (Italy). Alcian Blue and Periodic acid–Schiff (PAS) stains were used on all slides. Alician Blue followed by PAS stain can be useful in detecting glycosaminoglycans (Maffulli et al. 2000; Tallon et al. 2001), which are synthesized by fibroblasts (Roberts and Harding 1994) and facilitate wound repair (Trowbridge and Gallo 2002).

## Histology results

**Fig. 1:**
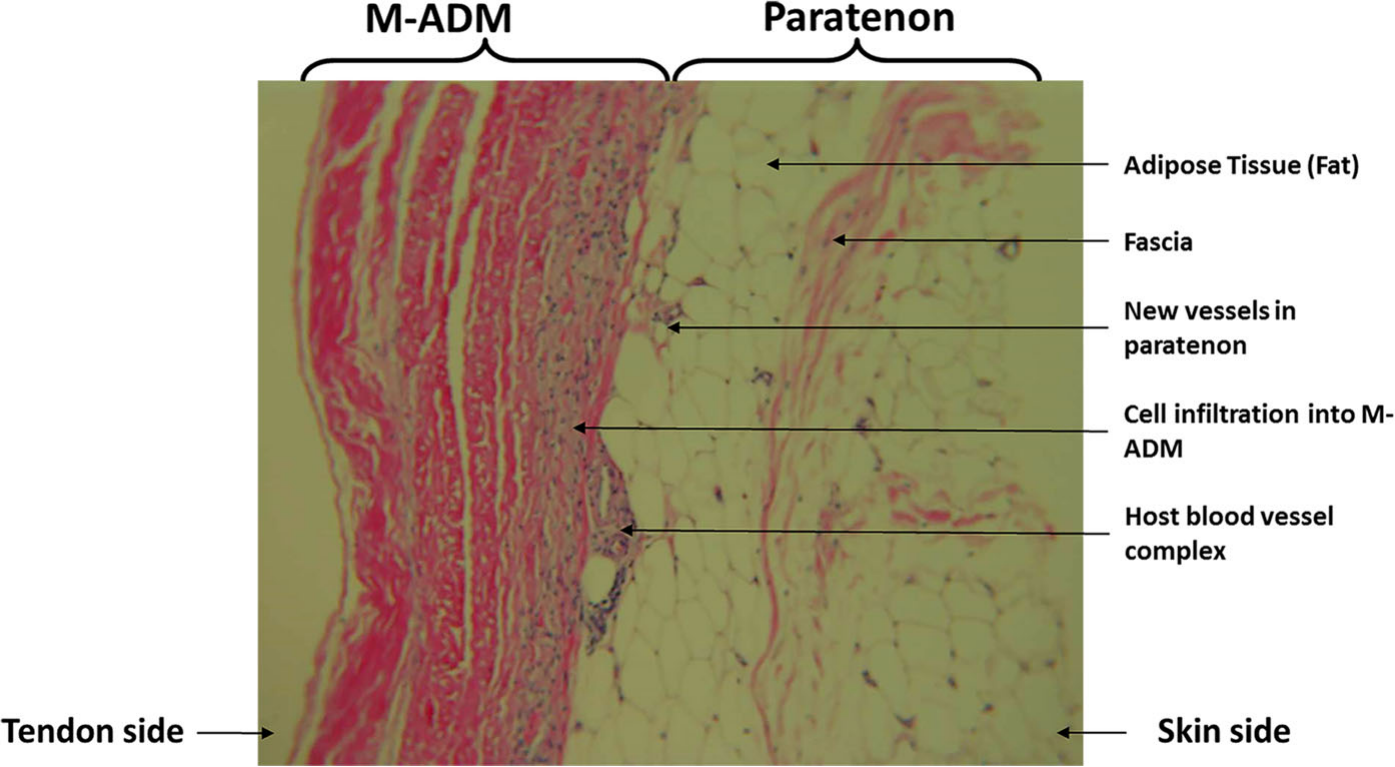
Graft-paratenon Interface—Alcian Blue/PAS Stain of Section 1 (low magnification) demonstrating host cell infiltration into M-ADM and new vessel formation in the paratenon

**Fig. 2:**
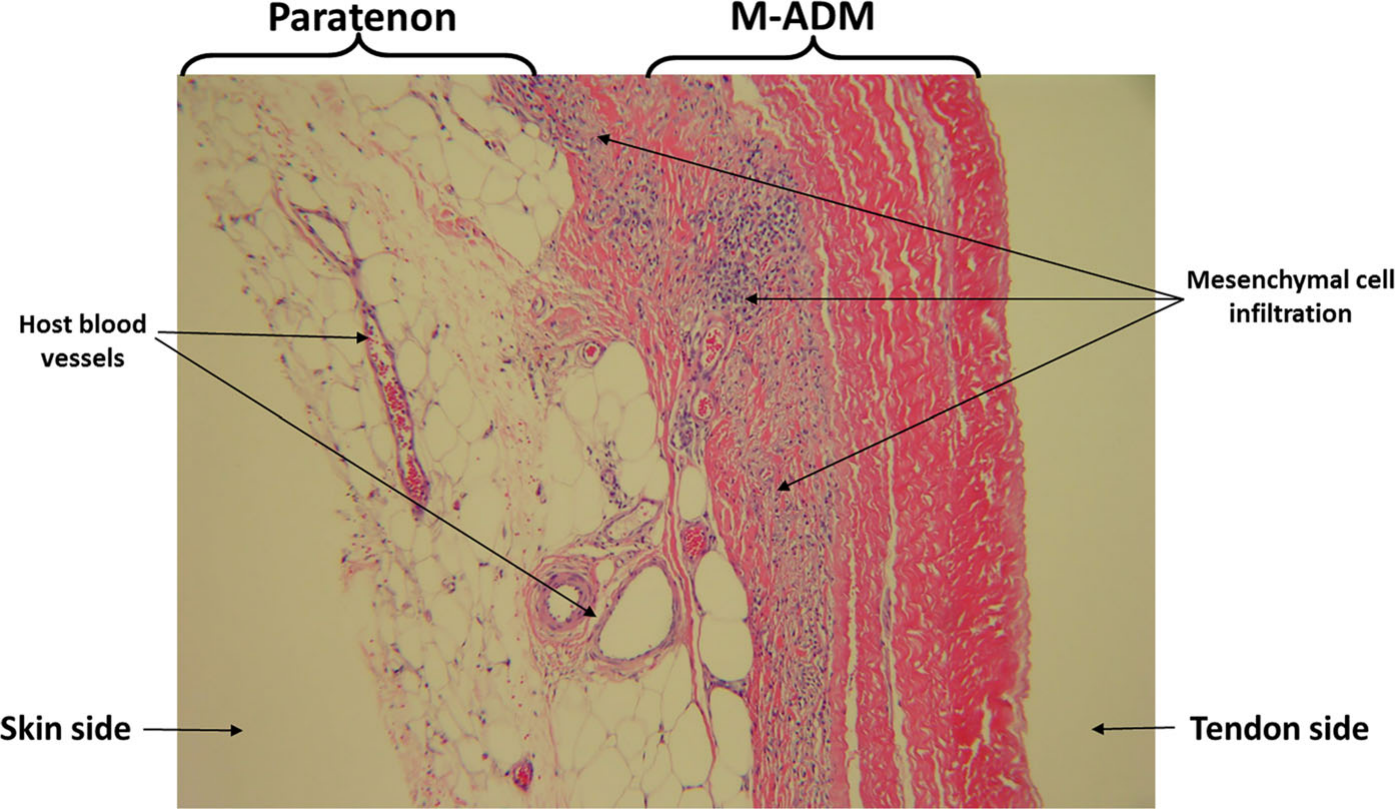
Graft-paratenon Interface—Alcian Blue/PAS Stain of Section 3 (low magnification) displaying increased blood vessel formation in the paratenon with mesenchymal cells infiltrating the graft

**Fig. 3:**
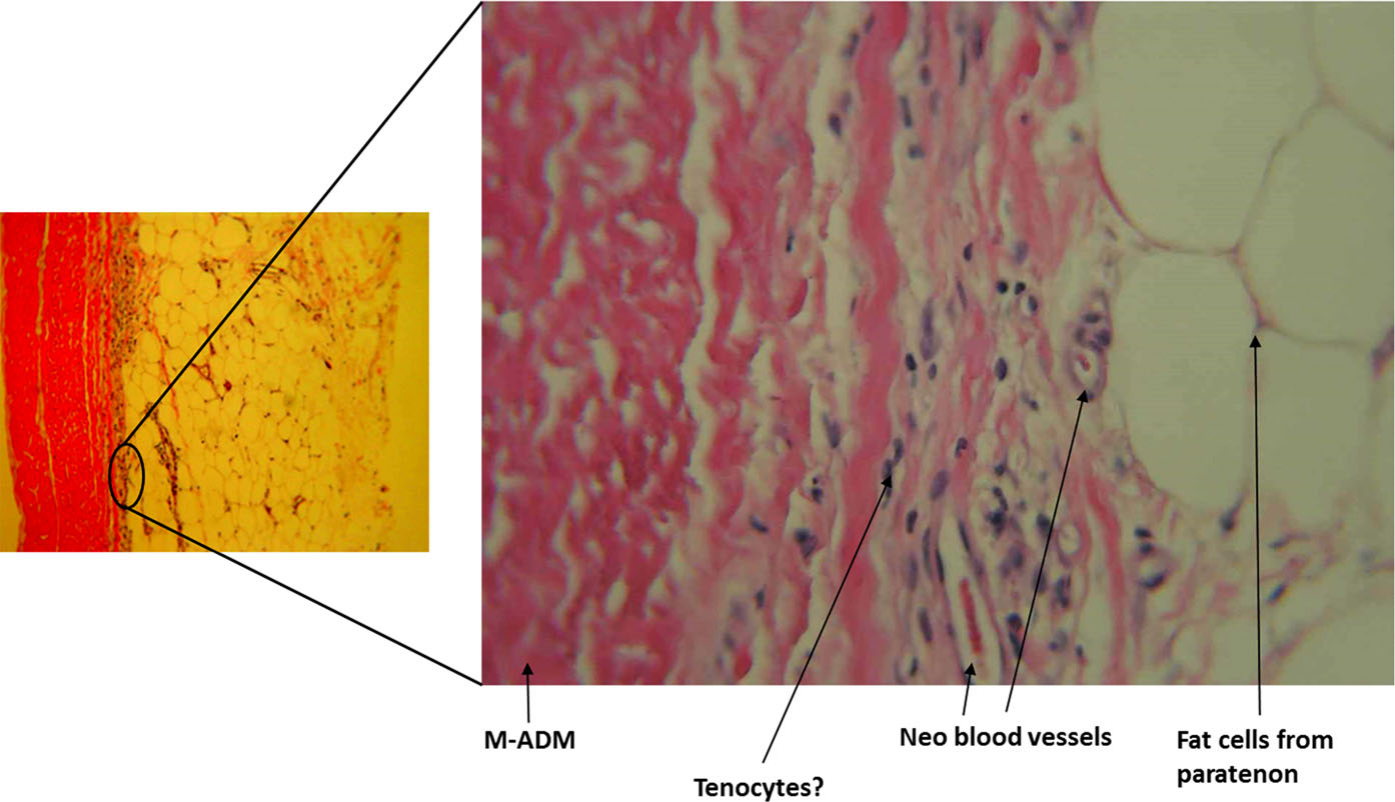
Graft-paratenon Interface—Alcian Blue/PAS Stain of Section 2 (hi magnification) showing tenocyte and neo blood vessel formation infiltrating the graft from the paratenon side

**Fig. 4:**
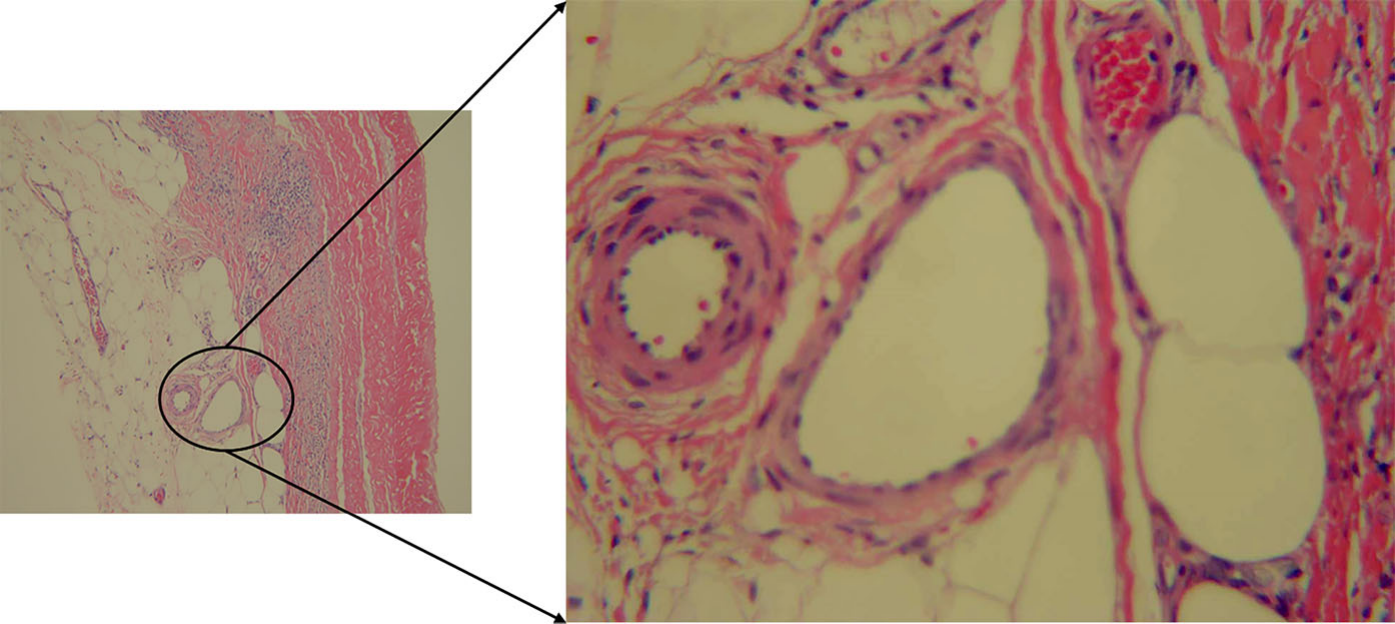
Graft-paratenon Interface—Alcian Blue/PAS Stain of Section 3 (hi magnification) depicting host blood vessel complex from the paratenon

**Fig. 5:**
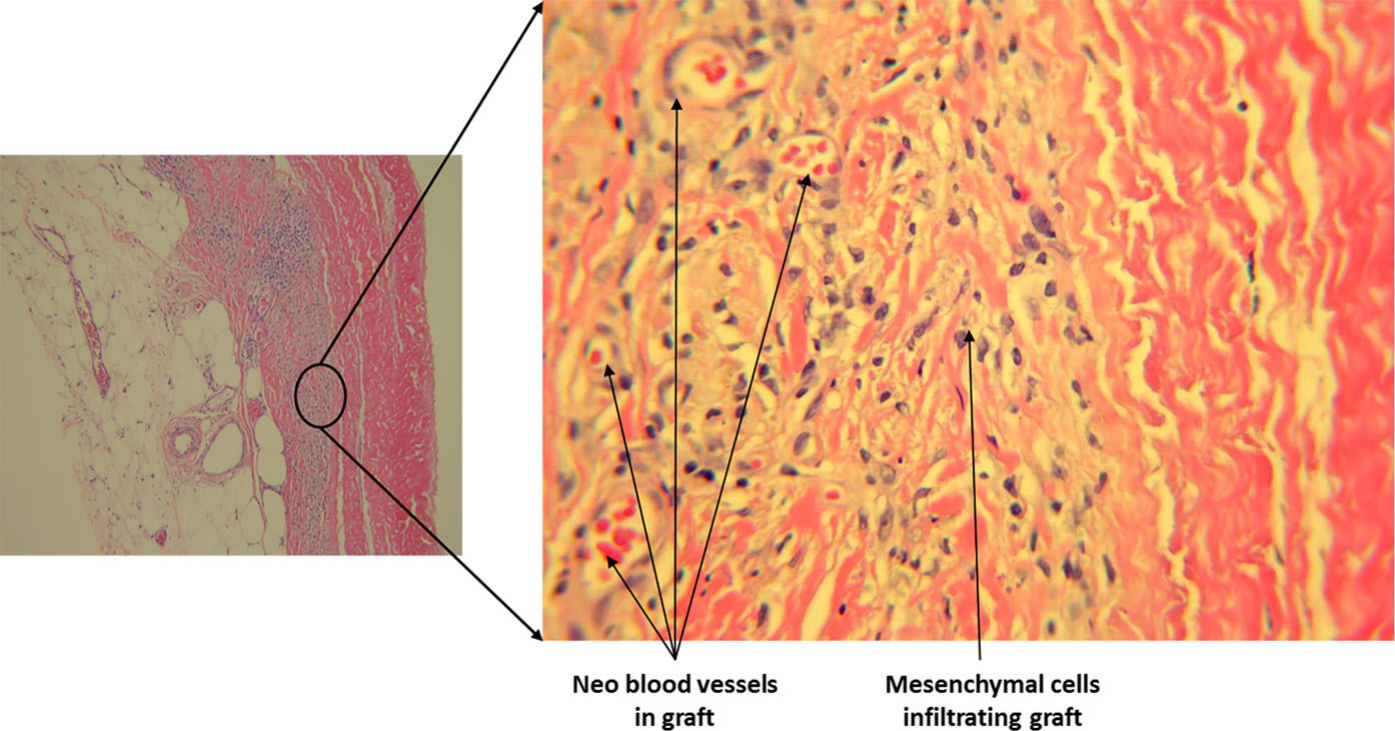
Graft-paratenon Interface—Alcian Blue/PAS Stain of Section 3 (hi magnification) demonstrating neo blood vessel and mesenchymal cell formations in graft

**Fig. 6:**
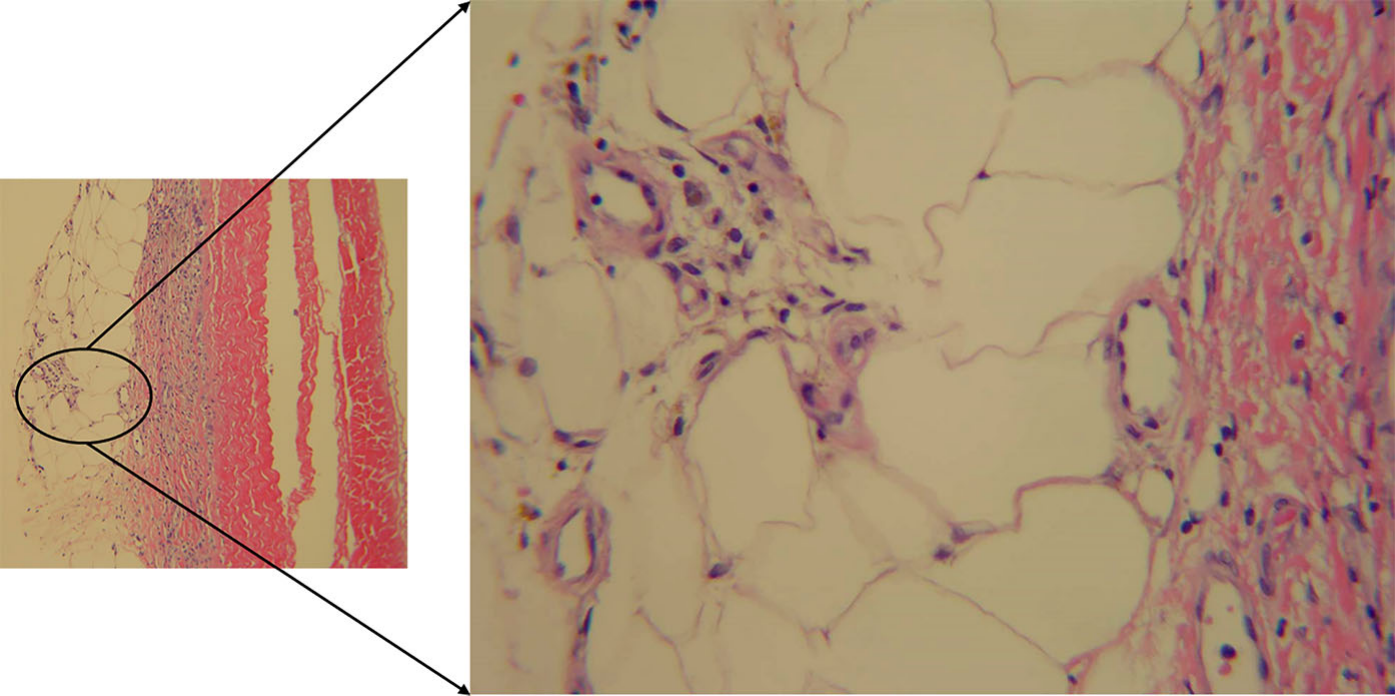
Graft-paratenon Interface—Alcian Blue/PAS Stain of Section 4 (hi magnification). Normal blood vessels and healthy host tissue (paratenon) can be seen around graft on left side. Formation of new blood vessels and cells were observed infiltrating the graft on the right side

**Fig. 7:**
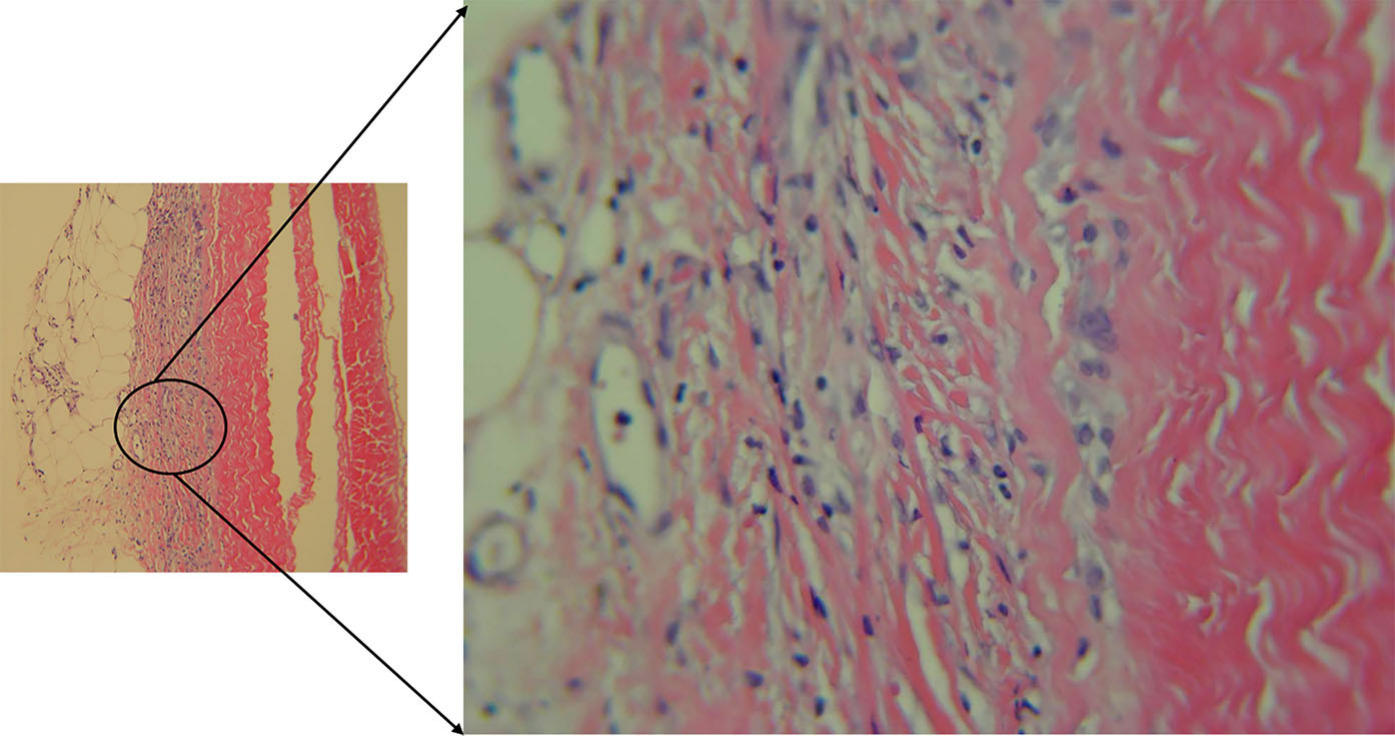
Graft-paratenon Interface—Alcian Blue/PAS Stain of Section 4 (hi magnification). Formation of new blood vessels on graft margins on the left side and host cells (mesenchymal type) infiltrating graft on the right side

**Fig. 8:**
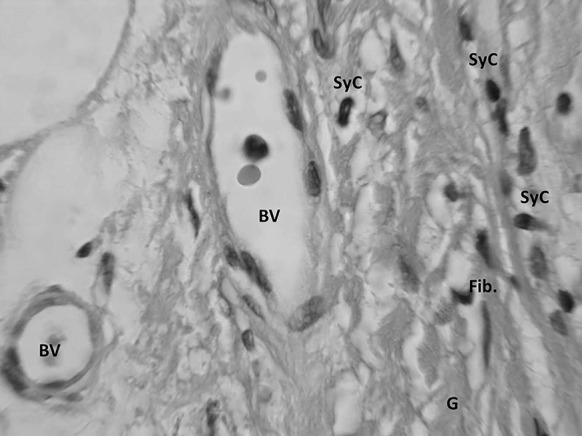
Graft Substance—Alcian Blue/PAS Stain. High magnification image of remodeling graft (G) showing appearance of new blood vessels (BV), and mesenchymal cells that are likely synovial (SyC) and fibroblastic (Fib) phenotypes based on morphology

**Fig. 9:**
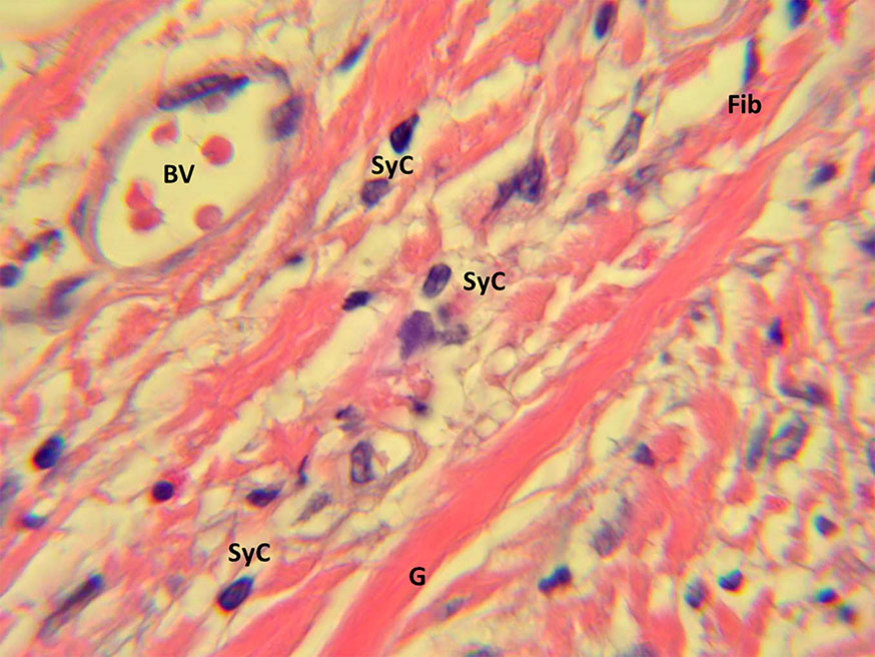
Graft Substance—Alcian Blue/PAS Stain. High magnification Image of remodeling graft (G) showing appearance of new blood vessels (BV), and mesenchymal cells of synovial (SyC) and fibroblastic (Fib) phenotypes based on morphology

Sections were taken at 8 weeks post-operative. All sections showed excellent attachment of paratenon to M-ADM with no evidence of any inflammatory response seen in any area. Low magnification images showed large areas of graft-paratenon interface (Figs. 1, 2) while the high magnification images concentrated on remodeling features (Figs. 3, 4, 5, 6, 7, 8, 9). Active infiltration of cells were seen from paratenon into graft (Figs. 1, 3, 6) and the infiltrating cells appeared mesenchymal (likely synovial based on morphology) in nature (Figs. 2, 7, 8, 9). Neo-vascularization was seen within cell infiltrated areas (Figs. 1, 5). Robust vascularization was also observed in the graft-paratenon interface (Figs. 2, 4, 7, 8). Revitalization of the graft, with new blood vessel and cell formations, was directional from the paratenon side and up to 60% of the graft depth appeared vitalized with new cells in some areas.

## Discussion

As expected, the remodeling was being driven from the direction of the paratenon. Typically, tenoblasts and tenocytes are the 90–95% of the total cellular elements of the tendon while the remaining 5–10% includes synovial cells of the tendon sheath on the tendon surface and vascular cells in the endo and epitenon (Kannus 2000). M-ADM demonstrated high levels of biocompatibility as evidenced by the absence of inflammation within the graft and host tissue, the infiltration of appropriate host cells into the graft matrix, and the presence of active vascularization within and around the graft. While these results are consistent with healthy incorporation demonstrated in the literature (Agrawal et al. 2012; Capito et al. 2012), it is important not to generalize the results of this case study. The following literature review is only intended to provide a greater understanding of the mechanism for incorporation and explain the histological processes observed in our ADM biopsies. Comparisons between ADMs are included in the literature discussion as this inclusion is critical to understanding the mechanism for incorporation. However, these comparisons should not be extrapolated to contrast the clinical performance of M-ADM with other ADMs in augmented tendon repair.

The recellularization process is complex and needs to strike the correct balance of cellular migration in order to promote healing while avoiding a destructive response (Orenstein et al. 2010). At the initiation of this process, histological analysis has shown a landscape of CD31 positive cells migrating to the implanted matrix. This indicates an influx of macrophages and monocytes and with this a resulting increase in Interleukin 1-β. Interleukin-1β is a key macrophage/monocyte cytokine that signals for the activation of fibroblasts which then produce collagen fiber, elastin, and other extracellular matrix components (Orenstein et al. 2010).

Orenstein et al. (2010) conducted an in vitro assay in which the levels of interleukin-1β were measured in vitro after culturing peripheral blood mononuclear cells (PBMCs) on three commercially available ADMs, AlloDerm^®^ (Acelity, San Antonio, TX), FlexHD^®^ (MTF, Edison, NJ), and AlloMax™ (Bard Davol, Warwick, RI). The authors noted the chemical signaling systems of various cytokines (particularly interleukin-1β) and growth factors, are able to navigate a fine line between wound healing and destruction. Furthermore, it was demonstrated that there was a statistically significant increase in interleukin-1β expression with FlexHD and AlloMax when compared to AlloDerm. The authors proposed that the different processing methods or possibly the biologic variation between donors for the three human ADMs tested caused differences in both inflammation and tissue integration. While the exact relationship was not clear, the authors suggested these data demonstrate a strong relationship between macrophages and the resulting cellular remodeling.

In continuing the investigation of these initial findings, Agrawal et al. (2010) explored this topic more deeply in an animal study by comparing the macrophage phenotype composition and resulting tissue remodeling of four different ADMs: M-ADM (also described in the study as DermACELL), AlloDerm, DermaMatrix^®^ (MTF, Edison, NJ), and Integra^®^ (Integra Life Sciences, Plainsboro, NJ). The M1 macrophage phenotype is correlated with inflammation and pathogen elimination while the M2 macrophage phenotype is associated with tissue repair, immunoregulation, and constructive tissue remodeling (Kadl et al. 2010; Mantovani et al. 2004). Substantial differences were noted between all 4 ADMs throughout the endpoint of 42 days post-implantation. The three human ADMs (M-ADM, AlloDerm, and DermaMatrix) started with a predominately M1 phenotype composition. AlloDerm remained predominately M1 while M-ADM and DermaMatrix transitioned towards a M2 phenotype with approximately equivalent compositions of M1:M2 phenotypes by Day 21. Conversely, the bovine cross-linked matrix, Integra, consistently showed a mixed phenotype infiltration without any significant difference between M1 and M2 at any time point. Integra also demonstrated the lowest number of infiltrating macrophages while M-ADM showed the greatest amount. These observations resulted in the human ADMs showing a bell curve shaped distribution of cell infiltration over time while Integra demonstrated an increasing trend of macrophage migration.

In a separately published but related study to Agrawal et al., Capito et al. (2010) also conducted a histological analysis on the healing process that focused on recellularization and revascularization using implanted M-ADM (also described in the study as DermACELL), AlloDerm, DermaMatrix, and Integra. Recellularization is a precursor to revascularization, so investigating both processes can lead to a greater understanding of the differences in matrix remodeling among these ADMs. M-ADM showed the highest degree of cellular migration/infiltration at all time points through Day 42 and the greatest cellular density at each time point except at Day 42. Conversely, AlloDerm consistently ranked among the least for cellular migration/infiltration and cellular density at multiple time points and magnification distances. Although the clinical significance of greater cellular migration and cellular density is not clear, the authors theorized that faster integration and more rapid matrix incorporation may reduce the risk of infection. In addition to demonstrating increased cellular density, M-ADM also displayed statistically significant greater vascular formation at Day 7 with almost double the number of vessels as the other ADMs. There was no discernable differences between the ADMs at Day 14 or Day 21, but M-ADM again had the highest number of vessels at Day 42 with statistical significance compared with Integra and DermaMatrix. Interestingly, Agrawal noted the higher revascularization rate for M-ADM corroborates the higher rececullarization rate and also the greater macrophage infiltration rate. The authors concluded that significant matrix integration differences exist between ADMs and it is likely due to dissimilar “biological activity.” This difference in biological activity may be attributed to the differences in tissue processing among the products.

Animal studies can provide initial insight, but more reliable data can be obtained from clinical studies. While clinical studies are more difficult to come by for this topic, a few studies have been reported that conducted a histological analysis on biopsies taken from implanted M-ADM used in breast reconstructions for breast cancer patients undergoing mastectomies (Bullocks 2014; Vashi 2014; Yu et al. 2016). Bullocks (2014) focused on the clinical outcomes in a ten patient case series which also included biopsies taken from human ADMs: M-ADM (also described in the study as DermACELL), AlloDerm, and FlexHD. At 6 weeks (42 days), the M-ADM biopsy revealed an infiltration of fibroblasts and revascularization that indicated incorporation had occurred. In contrast, AlloDerm displayed far fewer fibroblasts and blood vessels, while FlexHD demonstrated a lack of vasculature. The authors concluded that faster ADM incorporation, indicated by rapid recellularization and revascularization, could result in fewer complications in breast reconstruction surgery, though the results of this 10 patient (18 reconstructions) case series should not be generalized. While there was no comparator ADM in Vashi (2014), two dermatopathologists identified recellularization marked by fibroblasts, an abundance of elastin, and robust vasculature in biopsy samples of M-ADM (also described in the study as DermACELL) taken at 16 weeks post-implantation. Yu et al. (2016) carried out a study in which 48 ADM and non-ADM breast capsule biopsy specimens from 15 patients were histologically analyzed. An experienced and blinded dermatopathologist determined the ADM biopsies contained significantly less inflammation and fewer myofibroblasts than non-ADM capsules. The authors concluded that the decrease in myofibroblasts could be associated with decreased rates of capsular contracture. Similar to the two studies previously discussed, the authors suggested that differences in proprietary processing and sterilization of each ADM can attribute to the differences in cell population migration.

Understanding the mechanisms by which ADMs incorporate with host tissue plays an important role in increasing the strength and consistency of repairs. Furthermore, several studies have shown that different ADMs demonstrate varying degrees of recellularization, revacularization, and ultimately incorporation. Many of these authors have concluded that the different sterilization and manufacturing processes are responsible for the varying levels of integration displayed by ADMs. In studies where multiple ADMs were compared (Agrawal et al. 2012; Capito et al. 2012), M-ADM frequently demonstrated the greatest degree of integration as shown by the extent and onset of recellularization and revascularization. As suggested by some of the reviewed studies (Capito et al. 2012; Orenstein et al. 2010), the integration properties of M-ADM may be due to the unique process used to decellularize and sterilize the matrix. Notably, this process is proven by the removal of ≥97% of DNA from dermis, leaving the matrix with an average residual DNA level of 15.97 ng/mg dry weight (Moore et al. 2015). Other human ADMs have reported residual DNA levels of 134.46 ng/mg dry weight and 272.8 ng/mg dry weight, a respective increase in residual DNA of 840 and 1710% over M-ADM (Moore et al. 2015). Residual DNA content is an indicator of the thoroughness of the decellularization process which is important because cellular remnants in ADMs may hinder the healing process and promote a less desirable host response (Crapo et al. 2011).

Descriptions of the proprietary decellularization processes are not often readily available and it was difficult to find details of these processes for several of the human ADMs discussed in this paper. M-ADM tissue is decellularized through a patented process that involves the use of an anionic agent, such as sodium dodecyl sulfate, to solubilize the cellular components and then extract these materials (Wolfinbarger et al. 2004, 2008). AlloMax tissue undergoes the Tutoplast^®^ tissue process which uses osmotic and oxidative treatments, including 1N sodium hydroxide and hydrogen peroxide, to remove cellular components and potentially immunogenic structures (Powers and Linden 2016). DermaMatrix is decellularized using a 1 M sodium chloride solution under agitation for between 12 and 48 h and may also include simultaneous 0.1% Triton X-100 solution soaks (Truncale et al. 2010).

## Conclusions

The histological results from our single patient cannot be generalized but do corroborate the findings presented here from laboratory studies and clinical case series. While data from a larger patient population would be more beneficial, this would also be difficult to obtain in tendon repair procedures where a second operation is not standard practice. Our histological analysis was only made possible due to an accidental fall of the patient which made revision surgery necessary. The histological findings presented demonstrated that the remodeling of M-ADM in a tendon reconstruction procedure is similar to the matrix integration observed in a wound repair procedure.
